# Perspectives on the sustainment of a healthy vending initiative in a university setting: a reflexive thematic analysis

**DOI:** 10.1017/jns.2025.12

**Published:** 2025-03-05

**Authors:** Jane Dancey, Belinda Reeve, Alexandra Jones, Julie Brimblecombe

**Affiliations:** 1 Department of Nutrition, Dietetics and Food, Monash University, Clayton, Australia; 2 The University of Sydney Law School, Sydney, Australia; 3 Food Policy and Law, Food Policy, The George Institute for Global Health, UNSW, Newtown, Sydney, Australia

**Keywords:** Contract, Healthy vending, Private regulation, Sustained implementation, NCDs, non-communicable diseases, CFIR, Consolidation Framework for Implementation Research, HCGs, Healthy Choices Guidelines

## Abstract

The World Health Organization recommends countries adopt policies that encourage the creation of healthier food retail. In Australia, some organisations have created enforceable regulation for healthier food retail in settings under their contractual control. While progressive for public health, little evidence exists on the characteristics of individuals and organisations influencing sustainment of such initiatives. We explored the perspectives of those involved in a sustained (six year) real-world healthy vending initiative in a university setting in Melbourne, Australia. Qualitative interviews were undertaken with seven of the eight individuals involved in the initiative and informed by the Consolidated Framework for Implementation Research and the regulatory concept of social licence. Reflexive thematic analysis was used to generate themes on individual and organisational factors associated with sustainment. Two individual-level themes included participants enjoyment and skills for ‘getting the job done’ and working on innovative projects. Individual self-efficacy and enjoyment from working on innovative projects, combined with interviewees’ perception that their organisation had a role in leading social change, contributed to the initiative’s sustainment. Two organisation-level themes included the University leading innovation and having a responsibility to serve the needs of its community within the constraints of the need for ‘financial viability’ and the provision of ‘consumer choice’. This study brings to the fore evidence on the individual and organisational characteristics that contribute to the sustainment of a healthy food vending initiative from the perspective of those involved in implementation. Exploration of the importance of these characteristics to other food retail settings is required.

## Introduction

Consumption of unhealthy foods is associated with an increased risk of developing non-communicable diseases (NCDs) such as heart disease, stroke, diabetes and cancer which are the leading cause of death and illness globally^(1)^. As part of a suite of comprehensive strategies to address the growing burden of NCDs, The World Health Organization recommends that countries adopt policies to increase the availability, affordability and acceptability of healthier foods in food retail settings^(2)^. A small but growing body of research demonstrates that multiple inter-related factors promote the successful implementation of healthy food retail initiatives, including low cost of implementation, limited need for retailer input, maintenance of retailer profit making and ongoing consumer demand for healthier products^(3–7)^. However, there is less evidence on the factors influencing sustainment of healthy food initiatives^(7)^.

In Australia, several states have introduced policies or forms of regulation that apply compulsory healthy food and drink standards to food retail in certain settings such as hospitals and health services to increase the availability of healthy food and drinks to visitors and staff^(8,9)^. However, in the absence of government-led regulation to drive the transition to healthier food retail in most other sectors, some organisations have used forms of enforceable regulation, such as contracts to enable the creation of healthier food retail in the settings under their control^(10)^.

Vending is a food retail setting which has traditionally sold unhealthy food and drinks such as sugar sweetened beverages and ultra-processed, shelf-stable snacks high in fat, salt and sugar^(11)^. Globally, it has been subject to regulation, including the use of contracts to improve the availability and accessibility of healthy food and drink products^(10,12,13)^. There is little evidence however on the factors that promote the successful sustainment of such regulation.

Traditionally, policymakers have believed that regulation incentivises compliance and contributes to sustainment, particularly if non-compliance is linked to significant penalties, financial or otherwise^(14)^. More recently, regulatory scholars have identified that compliance with regulation may be more closely linked to the organisation’s ‘perception’ of risks rather than the actual likelihood of being penalised^(14)^. Research also identifies other motivators that encourage compliance with regulation that relate to the characteristics of individuals and organisations, including a general belief in the legitimacy of the regulation, perceptions of the social costs of non-compliance, shame or guilt and local community deterrents, e.g. when the local community has advocated for the regulatory compliance measure^(14)^.

The term ‘social license’ is used by organisations to describe how their activities may be controlled or modified by such societal expectations, regardless of whether they are required by regulation. A social license may also motivate organisations, and the individuals working for them, to act in ways beneficial to society and to guide an organisation’s decision making, strategic direction or to enhance its reputation^(14–18)^.

Whilst the literature offers insights into the characteristics of individuals and organisations that may influence the implementation of healthy food retail initiatives, there is less understood about the influence of these characteristics on healthy food retail initiatives implemented via contracts^(3,19–21)^. Further, many university campuses in Australia are ‘complex food retail settings’ with multiple food retail outlets and vending machines operating in close proximity which cater to the needs of students, staff and visitors^(22,23)^. There is little research examining the factors influencing the sustainment of healthy food initiatives in complex food retail settings like universities^(22)^. University food retail settings in Australia are often managed via contracts between the retailers and the university and therefore provide an opportunity for the university, as the contract owner, to include health promoting clauses in the contract.

In this paper, we examined a healthy vending initiative in a large urban university in Australia where health promoting clauses were included in a vending contract six years prior to the research being undertaken. Our aim was to elucidate the perspectives of those responsible for the initiative on why this initiative may have been sustained for over six years, focussing on the individual and organisational factors that contributed to its sustainment.

## Methods

### Study design

We used a qualitative design with single case analysis and the use of one-to-one interview data^(24)^. The Consolidated Framework for Implementation Research (CFIR) and the theory of social license informed a semi-structured interview guide. Braun and Clarke’s phases for reflexive thematic analysis was used to generate themes from the interview data^(15,25–28)^. The authors used a constructionist epistemology, rejecting the idea that there was an objective answer waiting to be discovered, to elucidate the individual and organisational factors that lead to the sustainment of the initiative from the perspectives of the participants^(29)^. This study was conducted according to the guidelines laid down in the Declaration of Helsinki and all procedures involving human subjects were approved by the Monash University Human Research Ethics Committee [Project ID 32142]^(30)^. Written informed consent was obtained from all subjects. Reporting of the research herein follows the Standards for Reporting Qualitative Research (SRQR, Supplementary file)^(31)^.

The CFIR is an analytical tool that can be used to examine the multiple different factors that may influence the implementation and sustainability of initiatives^(28)^. For this paper, we drew on the CFIR domain ‘Characteristics of Individuals’ as we were specifically interested in the individuals involved in approving or implementing the initiative and how they operated within a large and complex university environment^(28)^. The ‘Characteristics of Individuals’ domain describes five constructs relating to individuals: knowledge and beliefs about the intervention (the individual’s attitude and understanding toward the intervention); self-efficacy (the individual’s belief in their ability to perform the task); individual stage of change (the phase of change the individual is in and readiness for change); individual identification with the organisation (how the individual views their organisation and their commitment to that organisation); and other personal attributes (such as knowledge, skills, tolerance of ambiguity, motivation, values, learning style)^(28)^.

In addition, Gunningham’s theory of social license, which examines the extent to which organisations feel compelled, or constrained, to meet societal expectations, whether they are compelled by law, or not, was used to frame the interview guide to examine if there were additional societal factors that influenced the implementation and sustainment of the initiative which was implemented on a university campus^(15)^.

### Reflexivity and our research team

Our research team are practitioners and academics with research interests in public health nutrition, public health law and regulation. Author 1 is a dietitian, employee and PhD student at the large university where the initiative was implemented and led the implementation of the healthy vending initiative prior to commencing her PhD studies. Author 4 is a researcher at the same university with experience in co-design, implementation and evaluation of public health initiatives, however she was not employed by the university at the time the initiative commenced. Authors 2 and 3 are public health law academics with experience in public health regulatory initiatives and are not affiliated with the university where the initiative was implemented.

For this paper, we applied Braun and Clarke’s steps for reflexive thematic analysis to generate an interpretation of the individual and organisational factors that contributed to this initiative’s sustainment. This approach recognises the inseparable influence the researcher has on the generation of the themes from the data and does not attempt to neutralise the researcher’s perspective^(25,32,33)^. It also uses a flexible and iterative approach, compared to other more structured thematic analyses and involves ‘critical attention to personal, interpersonal, methodological and contextual factors that influence the study being conducted’^(27,32)^. Accordingly, Author 1’s position as an insider in this initiative is acknowledged and her understanding of the initiative, and the participants themselves, was considered inseparable from the analytic process. Taking this constructionist approach, we sought to understand the perspectives of the individuals involved in the approval or implementation of the healthy vending initiative^(34)^.

### Context

The setting for this initiative is Australia’s largest university, an urban university with four campuses in Victoria, Australia with over 50 food retail outlets and 100 vending machines, serving over 80 000 students and 17000 staff. In 2015, the university adopted a healthy eating policy which included voluntary initiatives to improve the healthiness of food and drink vending, catering and retail. In 2016, a nutrition consultant (Author 1) was employed within the Health and Wellbeing team to implement these initiatives. The university vending contracts were due for renewal in 2016, and the Victorian Government’s healthy vending guidelines, based on their Healthy Choices Guidelines (HCGs) were incorporated into the vending Expression of Interest and subsequent contract documents^(35)^. The HCGs state that at least 50% of vending products must be ‘Green’ or ‘best choice’ products with no more than 20% ‘Red’ or ‘limit’ products. The remaining products are ‘Amber’ or ‘choose carefully’. In practice, the vending machines contained 50% Green, 30% Amber and 20% Red products. In addition, all products were marked with coloured logos to indicate these choices. A sticker was displayed on the outside of the machine to denote Green as best (or healthiest) choice, Amber as choose carefully (moderately healthy) and Red as limit (unhealthy). A contract was awarded to a vending business, which is still in place over 6 years later.

### Participants and recruitment

The eight individuals involved in the approval or implementation of the initiative including a contract manager, lawyer, communications officer, leasing manager, policy owner, wellbeing manager, senior and executive managers, were identified by purposive and snow ball sampling and invited by email to participate in a voluntary in-person or videoconference (Zoom) interview. All but two of the interviewees were still employed by the university.

Given the inherently limited number of people who were involved in the project’s approval or implementation, we were guided by Malterud’s ‘information power’, a concept used to describe how the more information the sample holds relevant for the actual study, the lower the number of participants is required^(24)^. To ensure the sample size of our study design covered the five criteria for high Information Power, being (1) a narrow aim, (2) dense specificity, (3) applied and established theory, (4) strong dialogue and (5) a single case analysis our study aim was narrow and focussed on the perspectives of those involved in the approval or implementation of the initiative; our interviewees were all experts in their field, and had knowledge of the initiative we were studying which meant our interviews contained strong quality dialogue^(24)^. Author 1 also had established professional relationships with the interviewees which assisted with building rapport and added to the strength of the interview dialogue.

### Interview guide

The interview guide (Appendix 1) was informed by the CFIR interview guide and the theory of social license, with questions pertaining to the interviewee’s knowledge and beliefs about the intervention; self-efficacy; stage of change; identification with the organisation; and social license^(15,28,36)^.

### Data collection

Seven interviews were conducted by Author 1 in-person (n=5) and on Zoom (n=2) from November 2022 to February 2023. Interviews were based on the interview guide (Appendix 1) and audio recorded. One individual, an external legal consultant who drafted the vending expression of interest did not respond to three email requests for interview.

### Data analysis

Audio recordings were transcribed verbatim by an external provider and then checked for accuracy by Author 1. Where required, corrections to the transcript were made by Author 1. The six phases of reflexive thematic analysis (familiarising yourself with the data; coding; generating initial themes; developing and reviewing themes; refining, defining and naming themes and writing up) were applied^(25,37)^.

#### Phase 1: Familiarisation

Initially, in a process of data familiarisation, the audio recordings were reviewed and the transcripts read and re-read by Author 1.

#### Phase 2: Coding

Next, whilst re-reading the transcripts, Author 1 highlighted any segments of text that where relevant to the research question and transferred the text into a MS Word table, repeating this process for each interviewee^(25,38)^. During this process, Author 1 systematically and thoroughly generated initial semantic codes, based upon the surface meaning of the text for each text segment^(25)^.

#### Phase 3: Generating initial themes

All initial codes were then printed and cut by hand. Codes were moved around by Author 1 and grouped according to similar underlying concepts and formed the initial themes to answer our research question ‘what are the individual and organisational factors that led to the sustainment of a healthy food vending initiative in a large organisation?’ The initial four themes generated broadly answered this research question.

#### Phase 4: Developing and reviewing themes

Interview transcripts were then uploaded to NVivo and the text data mapped to the initial themes. During this process, two additional subthemes were generated for theme two.

#### Phase 5: Refining, defining and naming themes

Author 1 then refined and named the four themes and two subthemes with concise and informative names that present as statements and capture the predominant concept for each theme and subtheme^(25)^.

#### Phase 6: Writing up

Author 1 kept a reflexive journal throughout the study process, starting with post-interview notes and continuing throughout the analysis and writing of the paper. The journal documented ideas and challenges faced during analysis which guide a coherent analytical path and drafting of the manuscript.

## Results

The themes generated from the interview data related to the interviewees’ perceptions of the organisation (the university) they worked for, and to the interviewees’ perceptions of themselves and the teams they worked in or led in relation to the sustainment of the healthy vending initiative. Themes are detailed below, with relevant quotes to illustrate our findings.

### Theme 1: Our organisation leads

Interviewees believed that the healthy vending initiative was one of the first of its kind in Australia and that it led the way for other large institutions to implement similar initiatives. They expressed pride that their organisation’s implementation of healthy vending provided tangible evidence of implementation that convinced other large institutions with a complex food retail setting to implement similar initiatives.

I think it really made a change in … higher education because then everybody from the other universities was contacting us and asking, ‘How did you go about this? How did you guys do this?’ Yeah, I feel like it has been effective seeing it elsewhere and how quickly it moved from us. IV1

Interviewees also discussed the impact that an organisation the size and scale of the university could have when it took the lead on initiatives like healthy vending. Interviewee two felt that the university could, and should, take the lead on such initiatives even before governments made any regulatory changes, and in doing so demonstrate successful change.

They [the university] also have the ability to make changes because we’re a big place and so they also have the ability to make changes that are not necessarily government-imposed – that are imposed just at [the university] – to start everybody else doing similar things. IV2

### Theme 2: Our organisation has a responsibility to its community

Interviewees reflected that the university had a social license to operate by expressing a sense that the university had a responsibility to support the health and wellbeing of its students (particularly those living away from their parents), and its staff and visitors.

I know the university understands it has a moral obligation to support, guide, and encourage those students to be best academically …. best performing as people, and that’s why we have counselling, that’s why we have leadership programs – we’re creating lots of opportunities for those people to develop as people as well as academically. And so I see us supporting them in eating well is just one pillar but an important pillar of their life with us at the university. So, I think there’s an implicit understanding that we have a care responsibility. IV3

There was also a sense that healthy food vending was just one dimension of a broader health and wellbeing programme that was part of the university’s commitment to their community.

…we want to create a holistic, positive working environment from all perspectives: psychologically safe and supportive environment, right through to safe from a procedural perspective, and certainly from a food choices perspective on campus. IV4

#### Subtheme 2.1: Our responsibility to our community is bounded by financial viability

Although interviewees wanted to support the community they serve, they also expressed a pragmatic limit or boundary to this based upon the need to be financially viable. Interviewees conceptualised their concern about financial viability though the lens of the vending operator being able to provide their service. They felt that ‘the market’ or the vending operator themselves would be limited in their ability to deliver healthy vending if there was a lack of consumer demand and it was not profitable.

… like everything, [it] is a kind of commercial operation so if the vending machines aren’t successful, then I would imagine that the operators of those machines would be putting pressure on us to revert back to the more common, less healthy foods in those machines…… I’m pretty confident in saying if we got squeezed by both the consumer saying that they’re not happy with the choice, and commercially from the operators’ perspective, I think we would have had pressure on us to revert back to the old machines. IV4

#### Subtheme 2.2: Our responsibility to our community is bounded by providing consumer choice

A second subtheme was the notion that the university must provide consumers with both unhealthy and healthy food choices. This provided another boundary to the university implementing initiatives to promote healthy food and drink options.

I think you’ll have some that will like them, you’ll have some that won’t but they have a choice – there’s 50:30:20, so for the ones that don’t want what’s in there, you know, the ones that want the 20 percent that’s the red, there’s options for everyone. IV1

While committed to providing healthy and unhealthy food choices, interviewees expressed some apprehension regarding restrictions on food choices, particularly in that the university could be seen as acting as a ‘nanny state’ or unnecessarily restricting personal choice, which in turn could have negative repercussions for the implementation of the healthy vending initiative. Interviewees thought that because the healthy vending initiative did not ban unhealthy products, but decreased the number available, it was still providing consumers with a choice, and therefore would be broadly acceptable to everyone, including those who approved the initiative, and consumers.

My gut feeling as well, because, as you know, it doesn’t preclude them from buying that chocolate bar or whatever, so we’re not being the ‘nanny state’ but what we’re doing is just creating that visual indicator that the healthy choice is the primary choice, it’s the majority of the product, it’s at the eye level, and it grabs your attention ahead of a sugary hit. IV3

### Theme 3: I (or we) can get the job done

Theme three related to interviewees’ sense of confidence, or self-efficacy, in being able to implement the healthy vending initiative. Interviewees who implemented the initiative expressed a sense of being able to get the job done, and leaders who approved the initiative expressed a sense of confidence in their team’s ability to deliver on the initiative. The endorsement of executive leadership was also noted by interviewees who felt that such high-level backing would lead to a greater chance of successful implementation.

Honestly, I was confident because I knew that I was running it. If I had to rely on others, I wouldn’t be as confident but I knew what I wanted to achieve and I knew having the support from the executives and the Vice-Chancellor, I knew it was going to work, as in I knew that that would be implemented and I knew that small bumps in the road or hiccups, just would be pushed aside. IV1

Interviewees also described a non-ideological pragmatism in implementing the initiative – a sense that they were implementers of other people’s ideas and gained their reward from the actual implementation, regardless of what the initiative was.

I was just supportive of the plan and that’s it really. I can’t say that I was passionate about it or it was something that was high on my list because there’s so many other things that we deal with, but I feel like our job is to implement the things that other people are passionate about. IV2

Interviewees expressed confidence in implementing the initiative but were less sure of the initiative’s likelihood of longer-term success, particularly given the tradition of vending machines providing unhealthy food and drinks.

I was confident we would implement them no problem; I wasn’t necessarily confident on their success because it was a change, and … culturally I think those machines are seen as snack food when you’re having a break to get you over the hump, to re-energise more so than buying either lunch or a healthy snack. IV4

### Theme 4: I enjoy working on innovative projects

Theme four concerned the idea that working on the healthy vending initiative brought enjoyment or a sense of value to the interviewees. Interviewees said that being involved in this particular initiative held their attention and was enjoyable because it was new and tested conventional boundaries. The notion of ‘excitement’ was spoken about in terms of how it made the interviewee feel and also related to their sense of motivation to get the job done (Theme 3).

I mean, from a professional perspective, I think it was an exciting project to work on. IV7

## Discussion

This paper focussed on the individual and organisational factors that contributed to the sustainment of a leading healthy vending initiative in Australia that used a contract between a university and a vending provider. Individuals involved in the initiative demonstrated both self-efficacy and a desire to work on innovative projects, and were supported by an organisational culture that encourages leadership and recognises the importance of having a social license to operate. Despite their interest in supporting health and wellbeing, actions by those involved with the development and implementation of the initiative remained limited by concerns about financial viability (profitability) and the provision of customer choice.

Self-efficacy is described in the CFIR as an individual’s level of confidence in their ability to perform the task^(28)^. Individuals in our study expressed a high level of confidence and trust in their ability to get the job done and secondly, leaders expressed a high level of confidence in their teams to implement the healthy vending initiative. However, this self-efficacy did not extend to confidence in the longer-term success of the initiative. Damschroder et al., note that individuals who exhibit a high level of self-efficacy are also more likely to commit to implementation, even when problems arise, which was also expressed by our interviewees^(28)^. Leader empowerment and support for those implementing this initiative was also a key factor in its success. Leader empowerment of individuals and teams to perform tasks is known to positively influence project outcomes, as is the trust that leaders display towards their teams to deliver projects^(39)^.

Both leaders and those involved in implementation expressed a sense of pride and excitement in being involved in the initiative and the concept of providing a healthier food environment to the university community aligned with interviewees’ values. Previous research examining factors for successful implementation in healthy food retail interventions have cited food store owners’ knowledge, values, and personal support for community health as key^(21,40)^. Shared values, a sense of pride, and a commitment to the community’s health were similarly noted by our interviewees. However, where the interviewees in this study differ markedly from food store owners described in previous research was in their ability to take risks, including calculated financial risks, in circumstances where they would not be personally financially disadvantaged by an unsuccessful initiative. Although not explored with our interviewees, it may be easier for university employees to engage in innovative, but financially risky, projects without the same fear of personal financial consequences that a small business owner may have.

We also noted that the university, as an organisation, was perceived by interviewees as having a legitimate place in society to lead, be innovative, and act responsibly towards its community and society more broadly. These sentiments reflect the CFIR construct ‘individual identification with the organisation’ which describes how staff are more willing to fully engage in implementation if they view their organisation favourably^(28)^. Interviewees expressed a sense that they could, and should, be working in ways that were socially progressive and novel, because the university modelled that behaviour in its values, teaching and research. Whilst universities see themselves as leaders in innovation, and there is some literature reflecting this, the literature mostly references academic and research innovation^(41)^. Our study demonstrates that the value of innovation in a university setting extends beyond academics alone, to professional staff. Accordingly, the healthy vending initiative’s alignment with the university’s culture of innovation and community leadership also contributed to its implementation and sustainment.

A sense of social license, or working for the benefit of those in their community, including students, staff and visitors was found to be a shared value across interviewees. Gunningham and colleagues describe a form of social license where local communities or non-government organisations have the power to potentially damage the reputation of non-compliant organisations in ways that compel the organisations to comply with regulatory standards^(15)^. Whilst our interviewees perceived that they were operating with a social license, based upon their own perception of the community’s expectations and the potential for a positive impact on the university’s reputation; there was no suggestion that the university’s reputation would be damaged by not implementing, or non-compliance with, the healthy vending initiative. While interviewees expressed a strong commitment to a greater ‘social good’ that the university could or should provide to their community, our results confirm there were limits in how far this extended.

Thornton and colleagues^(14,16)^ describe how ‘economic license’ can override ‘social license’ in situations where it is not economically feasible to act in a way that fits with an organisation’s social license or ideals. They propose that organisations operate within a ‘license model’ where they must navigate a business path based on their (1) economic license (financial constraints) (2) regulatory license (legal obligations) and (3) their social license (expectations of employees and society)^(42)^. Despite those responsible for the initiative not being personally financially accountable for the initiative, our analysis demonstrated that there was still a financial boundary (economic license) to the social license described by interviewees, similar to that described by Thornton et al.,^(42)^.

A sense of responsibility to the community was also constrained by providing consumer choice. Interviewees expressed concern about restricting, or being perceived as restricting, choice in vending. Several interviewees noted that because the healthy vending initiative still allowed limited availability of unhealthy (Red) food and drink choices, it was easier to implement and sustain. The term ‘nanny state’ was used by interviewees as a label that they perceived would negatively impact on the success of the healthy vending initiative. ‘Nanny state’ is a term used in Australia, and elsewhere, to indicate an overly restrictive, or paternalistic, use of state (or organisational) power that undermines individual’s free choice or autonomy^(43)^. Public health researchers and ethicists describe how the term ‘nanny state’ has been used to build successful campaigns against public health initiatives and therefore, our interviewees, perhaps rightly, assessed that avoiding accusations of ‘nanny state’ behaviour and reframing the initiative within the language of ‘choice’ was important to the implementation and sustainment of the healthy vending initiative^(44–46)^.

Middel et al conducted a systematic review of the barriers and facilitators to the implementation of healthy food store interventions using both the CFIR and a systems innovation framework^(21)^. They identified that retailers expressed a desire to support health promoting interventions but that this support was influenced by the perceived costs, risks and benefits of any intervention in much the same way that our participants described having a responsibility to their community, but feeling bound by the financial viability of the initiative^(21)^. A sense of community and the health values of retailers has also been shown to be a strong motivator for the implementation of healthy food interventions^(21,40)^. Brimblecombe et al concluded from a study with remote community stores in Australia that healthy food retail initiatives to restrict marketing of unhealthy food may be more amenable to food retail contexts where there is a strong sense of social purpose and a close retailer-community relationship, than those where commercial interests primarily drive retail practice^(47)^. A recently published review of reviews by Gupta and colleagues described the large and growing body of evidence on factors influencing the implementation of healthy food retail interventions, but notes the need for further research to identify the factors associated with both sustainment and scalability of such interventions^(7)^. Our study contributes to this nascent research area by contributing evidence regarding the organisational and individual characteristics that are important for the sustainment of healthy food vending initiatives.

### Strengths and limitations

Our single case analysis provides novel insights into the sustainment of healthy food vending initiatives in the context of a specific university. The strengths of our study that provide for robust findings are that: we were able to interview all project participants, except the external legal consultant, thereby capturing the experience and views of those involved with the initiative’s initiation and implementation; the first author who conducted the interviews and analysis had deep knowledge of the initiative and its context and an established rapport with the interviewees, enabling for a robust interpretation of the data; and, the application of Braun and Clarke’s reflexive thematic analysis allowed for a transparent and unique interpretation of the interview data combined with the contextual knowledge of the first author. Lastly, the ongoing reflexivity applied in reflexive thematic analysis enhances the consideration of the applicability of our results to other settings^(32)^.

Our findings were set in the specific context of a university and in food and drink vending, however food and drink vending exists worldwide and usually involves a contract between a site owner and a vending provider, thereby allowing for nutrition standards to be incorporated Universities have characteristics that may differ from other food and drink vending contexts. They serve a number of roles in society, including contributing to societal good, advancement and innovation. These characteristics were highlighted by our participants as important to enabling the sustainment of the healthy vending initiative. Our findings therefore may apply to other organisations which already have, or can embed, innovation and a sense of social license into their workplace culture. This study therefore contributes to the broader discussion around sustaining healthy food retail initiatives, however applicability of its findings to other complex and dynamic food retail settings is untested. A further limitation with regards to generalisability of our findings to other settings including those similar to the setting our of study, is our small sample size. Whilst our study provides the experience and views of all except one involved with the university’s food and drink vending initiative, and Malterud’s concept of Information Power guided our study approach to ensure robust findings with our inherently small sample, we cannot guarantee the transferability of our findings to similar university contexts. Further validation of our findings with other populations is required before generalisability could be inferred.

### Conclusions

Our findings reflect the opinions and attitudes of a team of individuals who were involved in the sustainment of a real-world healthy food vending initiative for over six years. They provide insight into the level of individual self-efficacy and identification with the organisation that enabled this project to be sustained, and which along with leadership support, may be important factors in the sustainment of other healthy food retail initiatives that are implemented with a contract. Our findings also reflect an organisational culture that enabled this initiative to be sustained for over six years (and ongoing at the time of writing), and we suggest that other organisations which already have, or can embed, a sense of social license into their culture and policies may be able to leverage similar characteristics for sustained healthy food vending initiatives. In the absence of government action to create healthy food retail environments, the impact of local, organisation-led changes to food environments should not be underestimated. Further exploration by other researchers of the individual and organisational factors identified here and how they can be harnessed in other settings to sustain healthy food retail initiatives is warranted.

## Supporting information

Dancey et al. supplementary material Dancey et al. supplementary material

## Supplementary material

For supplementary material accompanying this paper visit https://doi.org/10.1017/jns.2025.12
