# Supplementary material for: Perspectives on the sustainment of a healthy vending initiative in a university setting: a reflexive thematic analysis
Source: J Nutr Sci. 2025 Mar 5;14:e23. doi: 10.1017/jns.2025.12 (PMC11894401; doi:10.1017/jns.2025.12)
Supplement: Dancey et al. supplementary material [file S2048679025000126sup001.docx]

Perspectives on the sustainment of a healthy vending initiative in a university setting: a reflexive thematic analysis

#### Appendix 1: Interview Guide

# Interview introduction

General introduction including information about the research, confidentiality and confirmation of agreement to participate. Framing of each participant’s role and why they were asked to be interviewed.

**Knowledge & beliefs about the intervention**

1. What was your role in introducing/approving/implementing the university healthy vending initiative? (EXPERIENCE)
2. What do you think about the effectiveness of the intervention at the university? (OPINION)
3. How do you think this initiative came to be in the first place? From your perspective, what were the motivators or the rationale for developing the initiative? (KNOWLEDGE)
4. Who was involved in the initiative and in what capacity?
5. How did you feel at the time about the plan to implement the intervention at the university? (FEELING)
6. Would you say those feelings were positive or negative? (FEELING)
7. In your opinion, how are the healthy vending contract clauses perceived by vending operators? (OPINION)
8. In your opinion, how are the healthy vending machines perceived by customers? (OPINION)
9. The vending program has now been running for 6 years….Why do you think the program is still going? (KNOWLEDGE)

**Self-efficacy**

1. How confident were you that the university would be able to successfully implement healthy vending? (FEELING)
   - Why do you say that?
2. How confident were you that your customers would accept healthy vending? (FEELING)
   - Why do you say that?
3. How confident do you think your colleagues felt about implementing healthy vending? (FEELING)
   - Why do you say that?

**Individual Stage of Change**

1. How willing were you to implement the intervention? (FEELING)

**Individual Identification with Organization**

1. Do you think the University has any obligation (social license) to provide healthier options in vending? (OPINION)
2. Did your personal opinions about implementing healthy vending match the University’s desire to implement healthier vending? (OPINION)
   - Did that matter to you?

**Other settings**

1. Do you think there are limits to using contract clauses to create healthier food retail environments/outlets? (OPINION)
   - Why do you say that?
2. What do you think about healthy food interventions being used in other settings at the University, for example, in food retail like cafes and food stores? (OPINION)
3. Do you have any thoughts on how it might work or under what conditions it might work to include healthy food and drink clause in the contracts for food retail (cafes, food stores etc)? (OPINION)
   - Why do you say that?

# CLOSE

# Thank participant for involvement in research, enquire about anyone else they think should be interviewed. Discuss documentation of the interview and if they’d like to review the transcript. Enquire if they are happy to be contacted again, if required. Thank them again.
